# Regarding "Multiple calcifying hyperplastic dental follicle (MCHDF): a case report"

**DOI:** 10.15171/joddd.2016.020

**Published:** 2016-06-15

**Authors:** Sonal Grover

**Affiliations:** Assistant Professor, Department of Oral Pathology, Chrishtian Dental College, Cmc, Ludhiana, Punjab, India

Multiple calcifying hyperplastic dental follicles (MCHDF) are disorders characterized by multiple unerupted teeth with abundant calcifications and rests of odontogenic epithelium in enlarged dental follicles.^1^Recently, I encountered a case of MCHDF and while reviewing the scientific literature, I came across a similar case report published by Jamshidi et al,^2^ published in the *Journal of Dental Research, Dental Clinics, Dental Prospects*.^2^ The authors erroneously declared their case report as the 11th case of MCHDF based on their wrong interpretation of the total number of cases reported till then. The two cases reported by Lukinmaa et al^3^ were limited in the same quadrant of the jaw, and hence, were declared by Cho et al^4^ as single hyperplastic dental follicles (SCHDF) and not MCHDF. Thus, the case report by Jamshidi et al^2^ should be suitably considered as the 9th case of MCHDF in the literature and not 11th in number.

The detailed summary of all the nine cases, including the case reported by Jamshidi et al^2^ is given in the Table  below.

**Table:** Summary of multiple calcifying hyperplastic dental follicles cases until the report of Jamshidi et al^2^

**No**	**Authors (Year)**	**Age/Sex**	**Impacted teeth (Total No.)**	**Type of Calcification**
**1**	Sandler et al^5^(1988)	15/M	2, 13-15, 18, 20, 21, 28, 29, 31 (9)	Type I
**2**	Gardner et al^6^(1995)	40/M	2, 11, 15, 27, 29, 31 (6)	Type II > Type I
**3**	Gomezet al^1^(1998)	15/M	2, 5-7, 11, 12, 15, 18-22, 27-31 (17)	Type I and Type II
**4**	Cho et al^4^(2010)	11/M	2, 6, 11,15, 18, 19,31 (7)	Type I and Type II
**5**	Cho et al^4^(2010)	14/M	2, 6, 11, 15, 18, 31 (6)	Type I and Type II
**6**	Cho et al^4^(2010)	11/M	2, 15, 18, 31 (4)	Type I
**7**	Cho et al^4^(2010)	15/M	2, 4, 13, 15, 18, 20, 31 (7)	Type I with Liesgang ring
**8**	Cho et al^4^(2010)	17/M	2, 15, 19 (3)	Type I and Type II
**9**	Jamshidi et al^2^(2013)	19/M	1, 2, 15-18, 22, 27, 31, 32, right and left maxillary distomolars – one each (12)	Type A and Type B

## Editor’s note

The concern raised in this letter was shared with a reviewer of the paper of Jamshidi et al.^2^ The reviewer stated that the statement by Jamshidi et al—“to date, 10 cases of MCHDF have been published worldwide”—was mainly based on the paper of Cho et al,^4^ in which the authors report five cases of MCHDF. Cho et al^4^ claim, in the beginning of the discussion section, that there were only five published cases of MCHDF before their paper, but continue to discuss, later in the text, that the two cases reported by Lukinmaa et al^3^ should be regarded as single calcifying hyperplastic dental follicle. Our reviewer stated that if the two cases of Lukinmaa et al^3^ are considered as single, not multiple, then the claim raised in this letter is reasonable.

## Competing interests

The author declares no competing interest with regards to authorship and/or publication of this article.

Table. Summary of multiple calcifying hyperplastic dental follicles cases until the report of Jamshidi et al^2^
